# Phylogenetic and Functional Structure of Wintering Waterbird Communities Associated with Ecological Differences

**DOI:** 10.1038/s41598-018-19686-3

**Published:** 2018-01-19

**Authors:** Xianli Che, Min Zhang, Yanyan Zhao, Qiang Zhang, Qing Quan, Anders Møller, Fasheng Zou

**Affiliations:** 1 South China Botanical Garden, Chinese Academy of Sciences, Guangzhou, China; 2 University of Chinese Academy of Sciences, Beijing, China; 3 Guangdong Key Laboratory of Animal Conservation and Resource Utilization,Guangdong Public Laboratory of Wild Animal Conservation and Utilization, Guangdong Institute of Applied Biological Resources, Guangzhou, China; 4 Ecologie Systématique Evolution, Université Paris-Sud, CNRS, AgroParisTech, Université Paris-Saclay, F-91405 Orsay Cedex, France

## Abstract

Ecological differences may be related to community component divisions between Oriental (west) and Sino-Japanese (east) realms, and such differences may result in weak geographical breaks in migratory species that are highly mobile. Here, we conducted comparative phylogenetic and functional structure analyses of wintering waterbird communities in southern China across two realms and subsequently examined possible climate drivers of the observed patterns. An analysis based on such highly migratory species is particularly telling because migration is bound to reduce or completely eliminate any divergence between communities. Phylogenetic and functional structure of eastern communities showed over-dispersion while western communities were clustered. Basal phylogenetic and functional turnover of western communities was significant lower than that of eastern communities. The break between eastern and western communities was masked by these two realms. Geographic patterns were related to mean temperature changes and temperature fluctuations, suggesting that temperature may filter waterbird lineages and traits, thus underlying geographical community divisions. These results suggest phylogenetic and functional divisions in southern China, coinciding with biogeography. This study shows that temperature fluctuations constitute an essential mechanism shaping geographical divisions that have largely gone undetected previously, even under climate change.

## Introduction

In a recent large-scale biogeographical analysis, Holt *et al*. identified a distinct Sino-Japanese realm extending from Tibet in the west to the major Japanese archipelago in the east^1^. According to Holt *et al*., the new Sino-Japanese realm lies between c. 25°N and 40° N in East Asia. In southern China, the Oriental/Sino-Japanese boundary lies between c. 113°–114° E contributing to the east part of southern China as Sino-Japanese and the western part of southern China as Oriental.

Multiple drivers have shaped the biogeographical regions of the world^2^. Generally, sharp changes in climatic conditions determine extant biogeographical boundaries^2–4^. Limited divergences occur in areas with abrupt climatic transitions^5^. Indeed, climate is a major determinant of present-day limits of species distributions^6^ and ecological turnover is higher between regions with dissimilar climatic features^7,8^. To predict responses to climate change, ecologists must understand the patterns of temperature variation, the mechanisms by which animals cope with this variation, and demographic and fitness consequences^9,10^. Because geographic breaks depend on environmental variation and how this affects species interactions, the joint study of environmental variation and community structure has the potential to provide a mechanistic bridge for linking geographical breaks and community structures under different scenarios of environmental change^11,12^.

To understand the nature of a geographical break or ecological differences, it may be relevant to examine the phylogenetic and functional structure of communities that are distributed across this spatial context. Phylogenetic studies mostly focus on divergence among co-occurring species^13,14^. Phylogenetic structure represents variation in evolutionary history among species, and is based on the evolutionary distance between species in a phylogeny^15^. Functional structure reflects variability in ecological attributes among species or individuals^16^. Functional studies mostly focus on the ecological niche because they arose in a given environment, associated with particular selective pressures. Phylogenetic fields are likely to be related to specific traits that reflect variation in large-scale habitat or climatic preferences^17,18^. Climatic tolerances are not randomly distributed across phylogenies. Species sensitivities to climate change are expected to be clustered across the phylogeny^19^. This phylogenetic cluster may be associated with a clustered functional structure with reduced functional alpha and beta diversity if the habitat is filtering a subset of functional traits^20^. This could occur either if functional traits have a phylogenetic signal^21^. Thus, the joint study of phylogenetic and functional structures is necessary to provide a mechanistic approach for determining co-occurrence.

By comparing the phylogenetic and functional structure of wintering waterbird communities, disentangling drivers of patterns, we address two questions: (1) Whether phylogenetic and functional geographical breaks can be demonstrated in southern China; and (2) how phylogenetic and functional structures associate with temperature dynamics. This study contributes to an understanding of the evolution of controversial geographical regions in East Asian and its associated factors.

## Results

### Phylogenetic structures across geographical regions

**Figure 1:**
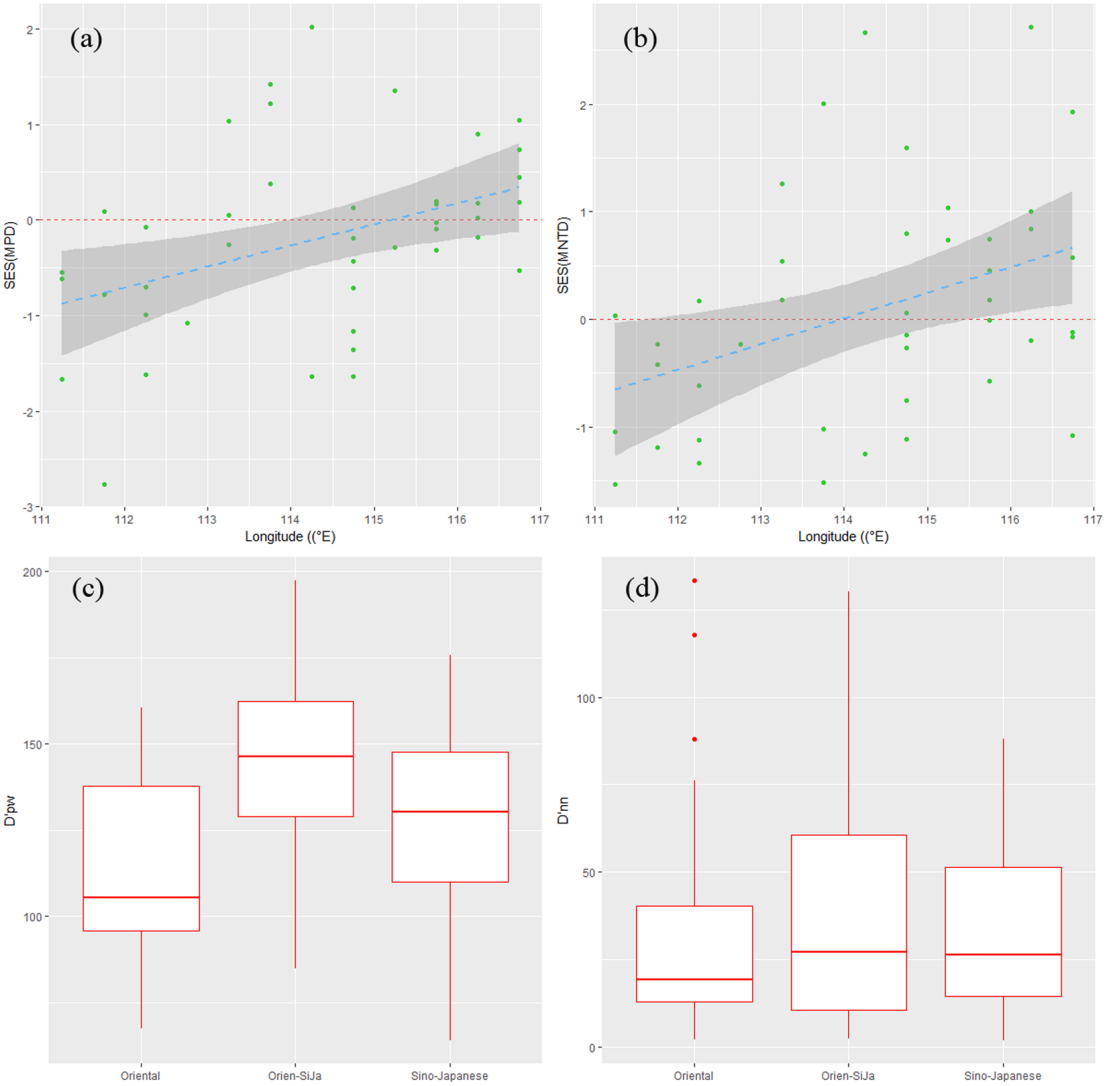
Patterns of phylogenetic structure for wintering waterbirds across geographical regions. (**a**) SES (MPD) and (**b**) SES (MNTD) beinging clustered at western zone (111°00′–112°59′) and overdispersion at eastern zone (115°00′–116°59′), fitted by GLM. (**c**) D′pw at the western zone being significantly lower than the eastern zone (*P*_adj_ < 0.01 and (d) D′nn at the western zone being lower than the eastern zone (*P*_adj_ = 0.55).

Phylogenetic structures of wintering waterbird communities showed clear geographical differences across regions. The trends shifted from cluster towards overdispersion from west to east (Fig. 1(a,b)). Specifically, SES (MPD) and SES (MNTD) were significantly different between west and east (both *P*_adj_ < 0.01, Table S1), dominant phylogenetic groups shifted from west to east, Charadriidae and Scolopacidae dominating in the west, Charadriidae, Scolopacidae and Anatidae dominating in the central part and Anatidae dominating in the east (see Supplementary Figs S1,S2).

The basal phylogenetic turnover (D′pw) differed significantly between regions (*P*_adj_ < 0.01, Fig. 1(c) and Table S1). The pattern of phylogenetic beta diversity also differed between west and the center when analyzing D′nn (*P*_adj_ = 0.02, Fig. 1(d), Table S1).

### Functional structures across geographical regions

Analyses of trait evolution showed that despite exhibiting a phylogenetic signal, traits tended to be evolutionarily labile because the resemblance between species was generally lower than expected under Brownian motion evolution (Table S2).

**Figure 2:**
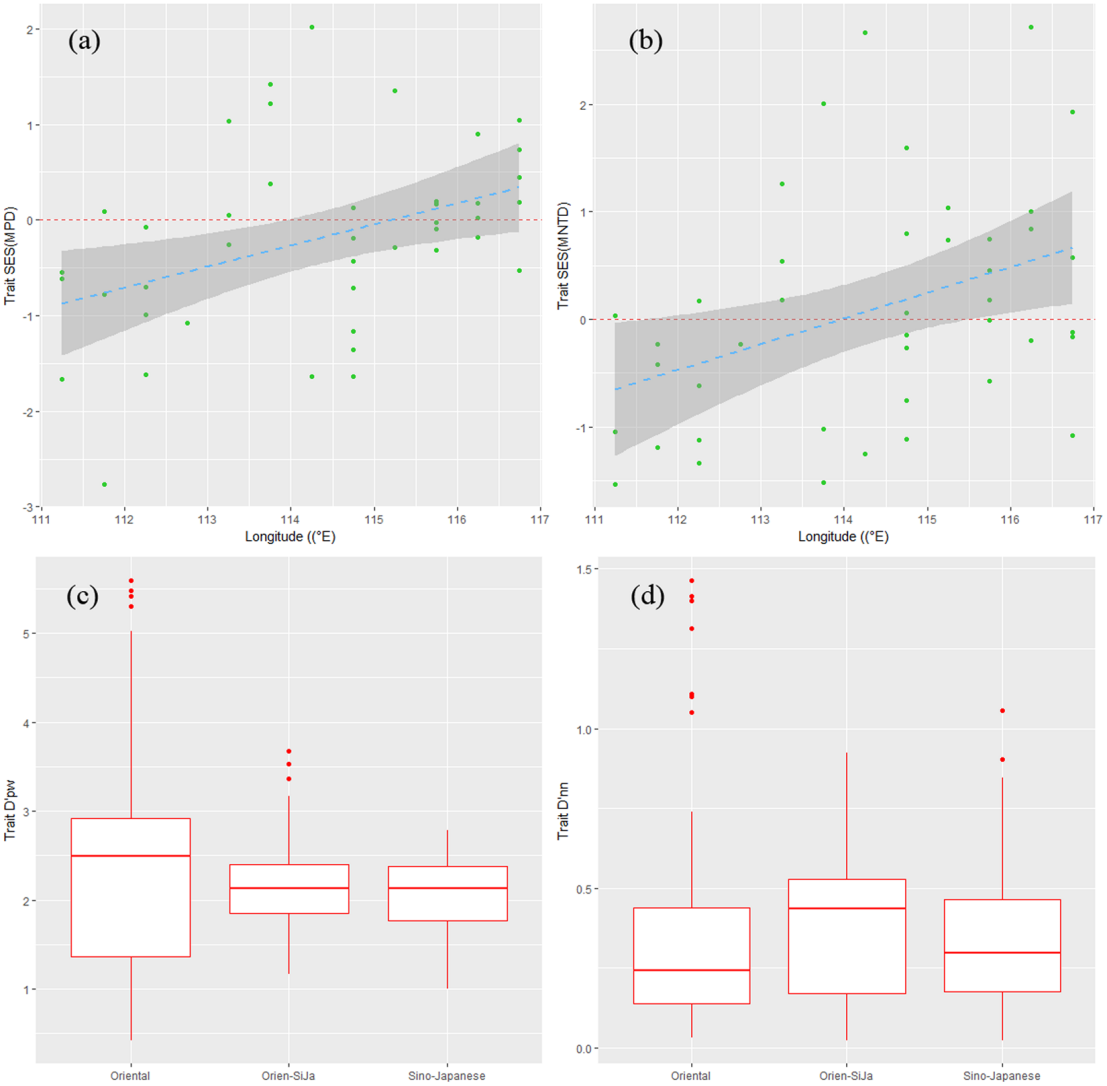
Patterns of functional structure for wintering waterbirds across geographical regions. (**a**) Trait SES (MPD) and (**b**) Trait SES (MNTD) showing cluster at west (111°00′–112°59′) and overdispersion at east (115°00′–116°59′),fitted by using GLM. (**c**) Trait D′pw at west showing significant higher than east (P_adj_ < 0.01 and (**d**) D′nn at west showing lower than east (P_adj_ = 0.19).

There were also shifts in dominant functional groups from west to east. Functional structure of waterbird communities also showed clear geographical trends across regions (Fig. 2). Specifically, Trait SES (MPD) increased towards overdispersion from west to east with a weak trend (Fig. 2(a)). Trait SES (MNTD) differed significantly between west and east, indicating that community functional structure shifted towards overdispersion from west to east (Fig. 2(b)).

Trait D′pw was lower in the west than in both center and east (both *P*_adj_ < 0.001, Fig. 2(c), while beta diversity did not differ from west to east when using trait D′nn (*P*_adj_ = 0.19, Fig. 2(d)).

### Drivers of phylogenetic and functional patterns

**Figure 3:**
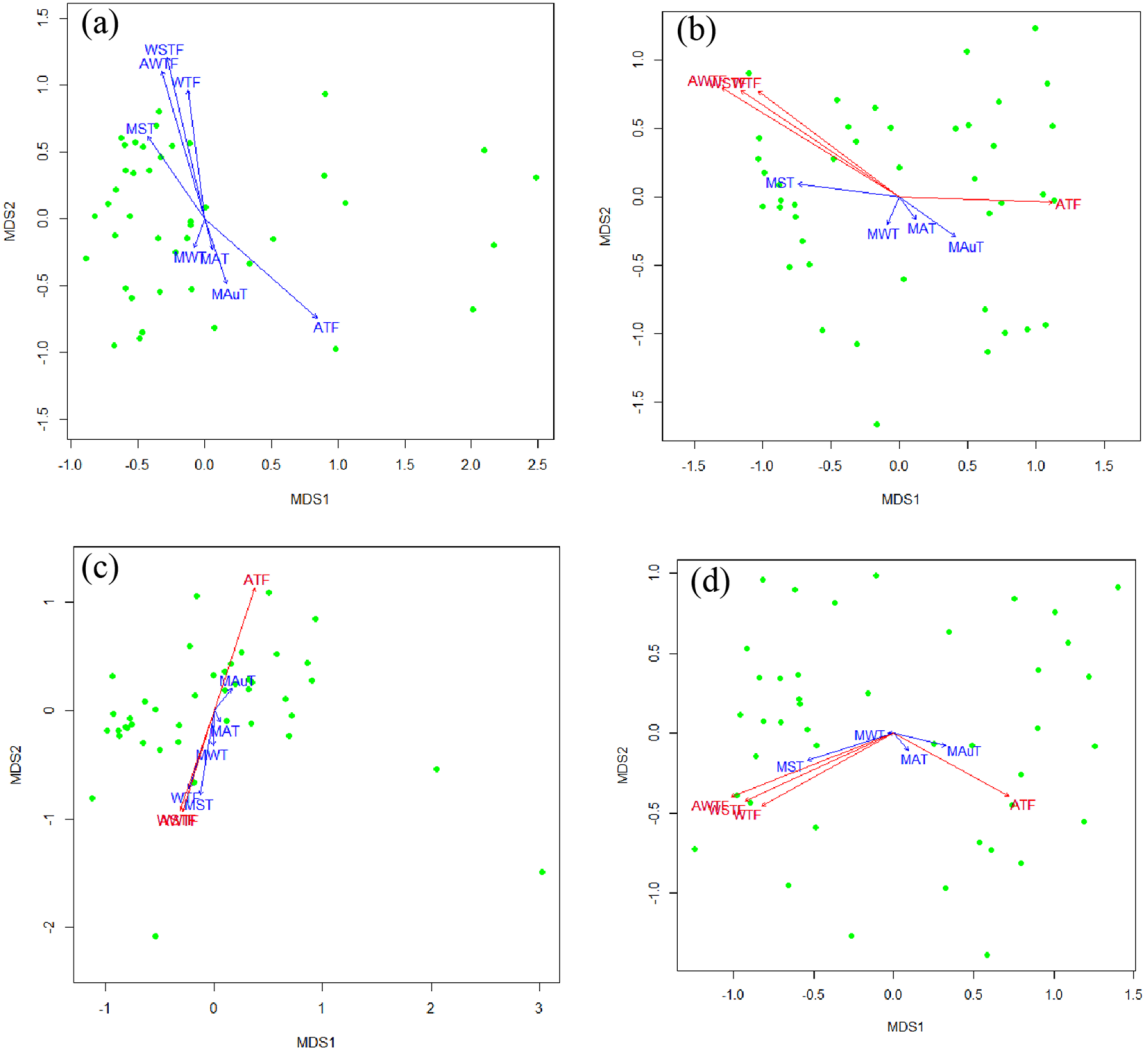
Temperature factors in ordination for (**a**) D′pw, (**b**) D′nn, (**c**) Trait D′pw and (**d**) Trait D′nn, respectively. Green points indicate wintering waterbird communities. Red arrows indicate significantly factors (p < 0.05) associated with a given measure, respectively. Blue arrows indicate factors associated with non-significant measures (p > 0.05), respectively.

When relating temperature factors to waterbird community dissimilarity, factors related to temperature fluctuation emerged as most important. Specially, WSTF (*P* = 0.05) was the most important factors for D′pw (Fig. 3(a)). ATF, WSTF, WTF and AWTF (All *P* ≤ 0.01) were the most important factors for D′nn (Fig. 3(b)). ATF, WSFT and AWTF (All *P* ≤ 0.02) were the most important factor for trait D′pw (Fig. 3(c)). ATF, WTF, WSTF and AWTF (All *P* ≤ 0.01) were the most important factors for trait D′nn (Fig. 3(d)).

## Discussion

### The effects of geographical differences on phylogenetic structures

Wintering waterbirds of southern China are a particularly valuable faunal assemblage that is suitable for testing general hypotheses about phylogenetic patterns and processes in mainland environments as a result of the unique geological and evolutionary history of the region. Using a comprehensive waterbird community dataset for southern China, we were able to demonstrate that phylogenetic patterns different between west and east. Overall, within-community waterbirds structure (SES (MPD) and SES (MNTD)) are significantly different between these two realms. The basal phylogenetic turnover (D′pw) of the western was significantly lower than that of the eastern part. Our results largely match the phylogenetic-based scheme of Holt *et al*.^1^, which suggest that the Sino-Japanese realm should be defined based on the phylogenetic distance that differ between Sino-Japanese and Oriental realms.

Even though Kreft & Jetz^22^ argued that only counts of branches, not their actual lengths, for quantifying dissimilarity will ignores considerable differences in the ages of clades and regions. Thus they recommended new approaches that account for all species in a single quantitative framework and that estimate branch lengths and uncertainty^22^. We investigated phylogenetic structures with distance-based metrics examining and contrasting the pairwise phylogenetic distances between species or distances for the nearest phylogenetic neighbor^23^. Those novel approaches can produce alternative, even refined, insights into the phylogenetic structure of assemblages. Accordingly, we confirm these geographical differences based on quality of data and choice of algorithm used.

### Effects of temperature fluctuations on phylogenetic and functional structures

The borders of biomes can readily be predicted from environmental variables^24^. Our regional study confirms the importance of environmentally determined differences. Thus, this separation should be described as an environmentally determined difference. Average temperature fluctuations compared to average temperatures emerged as sensitive determinants of phylogenetic and functional patterns in general. However, Ficetola *et al*. suggested that temperature heterogeneity is the strongest correlate of differences among these regions^2^.

Patterns of significant phylogenetic clustering and lower basal turnover (D′pw) in western waterbirds in the wetlands with greater temperature fluctuations suggest relatively harsher environmental conditions in parts of the west (Fig. S3). Climate fluctuation is acting as an ecological filter, only allowing a subset of species to colonize and survive. In the western region, an absence of Anatidae in most communities contributed to its clustering. Climatic tolerance variesy among species, causing some species to be more vulnerable to climate change than others^10^. The decline in beta diversity (D′pw) was associated with the decline in alpha diversity, because the same subset of species should be found in spatially disparate regions with temperature fluctuations^25^.

We also found shifts in the dominant functional trend across regions, with atransition from the west with Charadriidae with small body mass to the east with Anatidae with large body mass. Previous studies revealed that large species were the most clustered in broad-scaled bird co-occurrence patterns over evolutionary timescales^19^. Furthermore, environmental fluctuations filter for particular properties of size distributions whiles smaller organisms tend to be favored under warmer conditions^26,27^. As each individual must meet its metabolic demands, larger organisms will suffer disproportionately under rising temperatures because of their higher per capita metabolic rate^28^. Larger, longer-lived organisms with slower life cycles are most likely to face local extinction, because acute effects are the strongest when manifested within a single generation^29^. Increasing temperature fluctuations were alsoimportant as the increasing harshness of climatic conditions towards higher elevations is the most plausible explanation for the decrease in species richness at higher elevations^30,31^.

Incorporating temperature fluctuation into our understanding of wintering waterbird community structure and turnover has already been shown to further our understanding about the drivers for geographical patterns. Therefore, temperature fluctuations can be important when predicting the geographical distribution of all birds.

### The relationships between phylogenetic and functional structures

Our findings indicate that traits tended to be evolutionarily labile with a significant phylogenetic signal. Furthermore, phylogenetic basal diversity (D′pw) and functional basal diversity (trait D′pw) were positively correlated, and this pattern seems to be driven by taxon diversity: the relationship between D′pw and trait D′pw was not significant when we controlled for taxon diversity (TD, Table S4). However, this is in agreement with the results for phylogenetic signal, which displayed similar weak niche conservatism across regions. Consequently, a weak correlation between D′pw and trait D′pw is expected^15^. Thus environmental conditions among geographic regions that promoted different rates of trait evolution and speciation^32,33^.

However, the mechanism that drives the consistent relationship between phylogeny and function across geographic regions remains unclear. It is possible that the same result could occur in different regions independent of the mechanism. For instance, the stress-gradient hypothesis^34^ states that regional phylogenetic or functional diversity is filtered to a greater degree in local communities located in abiotically harsher than in benign habitats. If harsh conditions are not filtering phylogenetic or functional diversity components at the same rates, the relationship between phylogeny and function would be untenable.

## Conclusions

Using a novel phylogenetic perspective, we provide evidence that geographical patterns differd between wintering waterbirds. Geographical patterns exhibited consistent deviations that suggest the existence of structuring mechanisms, with structure being clustered in the west and overdispersion in the east. Less temperature fluctuations in the east may create more expansive niche space that can support viable populations and may allow for greater niche differentiation in these habitats. In contrast, the western part has less structural complexity, and represents inhospitable and stressful conditions, which could result in physiological constraints leading to competitive exclusion in which better adapted clades out compete other clades for limited resources.

## Methods

### Study area

This study was carried out in southern China prefecture (21°30′ N, 111°00′ E to 23°50′ N, 116°59′ E) located across the geographical break of the Oriental/Sino-Japanese realms. This area is also located within the East Asian-Australasian Flyway and harbors a large fraction of global waterbird diversity in its coastal wetland landscape^35–38^. These species are migrants that breed across the northern temperate belt from the Atlantic to the Pacific coastal wetlands. The wintering distributional ranges of those species cover major areas of Southern China and extend across the boundary between the Oriental and Sino-Japanese realms^35^. Like many parts globally, changes in climate could dramatically change the biodiversity landscape in this area^39,40^, making it a pressing need for understanding the implications for biodiversity. Community composition of wintering waterbird has not been well characterized in the region, with (to our knowledge) only two thorough waterbird surveys performed to date^41,42^.

Sites were assigned to different geographic regions using a map provided by the Geospatial Data Cloud (http://www.gscloud.cn/). Communities were found in one of three regions: west (Oriental, 111°00′–112°59′), center (Orien-SiJa, 113°00′–114°59′) and east (Sino-Japanese, 115°00′–116°59′).

### Bird surveys

Bird surveys took place during winter 2014 (December 2014 to January 2015), when the most-contrasting weather conditions occur in local coastal wetlands. Birds were counted in 42 coastal wetlands in Southern China (Fig. S1). The duration of surveys among wetlands (distance less than 20 km) were restricted to one day. We also restricted the duration of the survey to minimize errors attributable to inter-site movements. The “look-see” counting method was used, in which we selected a suitable vantage point and subsequently counted all visible waterbirds^43^. We used 12 × 50 binoculars to locate birds, and a hand-held GPS unit with a 5-m error to measure distances within and among wetlands. Scientific nomenclature and taxonomic sequence follow Jetz *et al*.^44^.

### Building the avian phylogenetic tree

Sets of 10,000 pseudo-posterior samples of phylogenetic trees were downloaded from http://birdtree.org/. We subsampled 1000 ‘Ericson All Species: a set of 10,000 trees with 9993 OUT each’ trees pruned for our full set of species and using the tree developed by Hackett *et al*. as a “backbone”^45^. From these 1000 trees, we calculated a maximum clade credibility tree using mean node heights with the software TreeAnnotator of the BEAST 2 package for use in subsequent analyses^46^.

### Functional traits and phylogenetic structure of waterbird communities

Four functional traits were selected to calculate functional diversity using a Gower dissimilarity matrix. The functional traits selected reflected morphological, physiological, reproductive, and phenology characteristics of each species. Although using traits to characterize the functional roles of species undoubtedly misses some aspects of the ecologies of species, direct links have been demonstrated between functional traits and diet, foraging pattern, and habitat preference^16,47^. For example, beak length and tarsus length were used to represent food capture, resource acquisition and allocation strategies of species, which are directly associated with competitive ability^48,49^. Trait values were obtained from the *Birds of China* and the online database “The Handbook of the Bird of the World (http://www.hbw.com)” using information for populations that occur in our study area whenever possible. Mean trait values for species were used for continuous traits. In this study, we collected traits measured to the nearest mm (potential waterbird wing length, beak length, and tarsus length) and body mass measured to the nearest gram.

To assess whether the four continuous traits were evolutionarily conserved or labile in waterbirds, we conducted a test using the multiPhylosignal function in Picante. We first employed a randomization test for phylogenetic signal^50^, which calculates the variance of the independent contrasts of each trait across the phylogeny and compares it with a null distribution of the variance of trait’s independent contrasts obtained from 1000 randomizations of the traits among species. Observed variances lying on the first or last 25 quantiles of the 1000 randomizations were considered evidence of significant phylogenetic signal or antisignal, respectively. We then used the K statistic to quantify the strength of the phylogenetic signal of traits relative to signal expected for traits evolving under Brownian motion. If K equals zero, differences in traits between species are proportional to the branch lengths separating them on the phylogeny. If K is greater than one, then traits are considered conserved because close relatives are more similar than expected under Brownian motion evolution; and if K is lower than one, then traits are considered labile.

### Functional and phylogenetic alpha diversity

We calculated MPD and MNTD among species in each community^15,51^. We performed this analysis using abundance-based data. We used an identical framework to calculate trait MPD and trait MNTD. Furthermore, we compared these indices to null models to test whether the phylogenetic and functional structures differed from random expectations. Specifically, we made the identities of those species random draws from the whole species pool, and then maintained the species richness of each community to generate random communities. The standardized effect sizes (SES) of MPD, MNTD, MFD, and MNFD were calculated as

1 $${\rm{Standardised}}\,{\rm{effect}}\,{\rm{size}}={{\rm{X}}}_{{\rm{obs}}}-{{\rm{X}}}_{{\rm{null}}}/{{\rm{SD}}}_{{\rm{null}}}$$

where X_obs_ is the observed value of MPD, MNTD, trait MPD or trait MNTD, X_null_ is the simulated values and SD_null_ is standard deviation of the simulated values. SES (MPD) and SES (MNTD) are equivalent to -1 times the net relatedness index (NRI) and the nearest taxon index (NTI), respectively. Positive SES values suggest phylogenetic or functional overdispersion, while negative values indicate clustering.

MPD is more sensitive to tree-wide distributions of lineages, whereas MNTD tends to be more sensitive to the distribution of lineages close to the tips of the tree^15,52^. The measures MPD and trait MPD represented “tree-wide” clustering and evenness, respectively, and phylodiversity and functional diversity were measured by MNTD and trait MNTD as indices of clustering and evenness toward the tips of the tree. Processes including dispersal, habitat specialization, and speciation have very different predicted effects on phylogenetic and functional diversity depending on how they are measured. These processes occurred in the distant past, and therefore effects on the entire clade of organisms are indicated by non-random patterns of MPD or trait MPD, whereas more recent processes, such as those during the past several million years, are indicated by significantly non-random patterns of MNTD or trait MNTD.

### Phylogenetic and functional beta diversity

Phylogenetic and functional beta diversity were calculated between pairs of plots belonging to the same or to different regions, to assess geographical turnover in phylogenetic and functional diversity. Both phylogenetic and functional beta diversity were calculated using two distance-based measurement metrics, the abundance weight pairwise distance metric (D′pw and trait D′pw), and the abundance weight nearest neighbor distance metric (D′nn and trait D′nn)^23^.

2 $${\rm{D}}^{\prime} \mathrm{pw}={\sum }_{i}^{n{k}_{1}\,}{\sum }_{j}^{n{k}_{2}}{\delta }_{ij}\,{f}_{i}{f}_{j}$$

3 $$\,{\rm{D}}^{\prime} \mathrm{nn}=\frac{{\sum }_{i}^{n{k}_{1}}\,min{\delta }_{i{k}_{2}}\times {f}_{i}+{\sum }_{j}^{n{k}_{1}}\,min{\delta }_{j{k}_{1}}\times {f}_{i}}{{\sum }_{i}^{n{k}_{1}}\,{f}_{i}\times {\sum }_{j}^{n{k}_{2}}\,{f}_{j}}$$

for D′pw and trait D′pw, where δ_*i*_ is the mean pairwise phylogenetic or functional distance between species *i* in community k_1_ to all species in community k_2_, and δ_*j*_ is the mean pairwise phylogenetic or functional distance between species *j* in community k_2_ to all species in community k_1_, and ƒ_*i*_ and ƒ_*j*_ are the relative abundances of species *i* and species *j*. For D′nn and trait D′nn, where min ${\delta }_{{{ik}}_{2}}$ is the nearest phylogenetic or functional neighbor to species *i* in community k_1_ to community k_2_, and min ${{\rm{\delta }}}_{{{jk}}_{1}}$ is the nearest phylogenetic or functional neighbor of species *j* in community k_2_ to community k_1_, and ƒ_*i*_ and ƒ_*j*_ are the relative abundance of species *i* and species *j*.

A pairwise distance metric generally reflects the overall dissimilarity between communities, while nearest neighbor distance metric is likely better for qualifying the patterns among close related species between different communities^23^. Null model communities for analyzing functional and phylogenetic beta diversities were generated by randomly shuffling richness cross the tips of phylogenetic tree or the traits matrix for 999 times^23^. Next, we used partial Mantel tests to calculate the correlation coefficients for pairs of dissimilarity matrices. Statistical significance was calculated with a Mantel Carlo permutation test using 999 permutations^53^.

### Temperature variables correlated with the community structures

Based on the knowledge on the main climatic drivers of the wintering waterbird distributions, we selected eight temperature variables as potential explanatory variables for wintering waterbird community’s structures: (1) average temperature, (2) temperature fluctuation. The average temperature predictors included mean annual temperature (MAT), mean autumn temperature (MAuT), mean wintering temperature (MWT) and mean spring temperature (MST).The average temperature conditions for each 0.5°-grid cell were derived from the China Meteorological Data Sharing Service System (http://data.cma.cn). Temperature fluctuation predictors included annual temperature fluctuation (ATF), autumn-winter temperature fluctuation (AWTF), winter temperature fluctuation (WTF) and winter-spring temperature fluctuation (WSTF) which were calculated according to monthly temperature.

We used adequate dissimilarity measures (Function metaMDS in the vegan library) recommended in community ordination to measure how temperature variables are correlated with phylogenetic and functional dissimilar^53^. Accordingly, we performed envfit function to get the p-value of correlation of each variable with overall waterbird communities. We performed two-dimensional nonparametric multidimensional scaling (NMDS) to plotting ecological correlates in the ordination to visualize the relationships among the communities and the determinant factors as recommended by Minchin (1987)^54^. The temperature vectors give the direction cosines which are the coordinates of the heads of unit length vectors. These “weak” predictors have shorter arrows than “strong” predictors. Then, we scaled relative lengths using command scores.

One-way ANOVA was used to analyze whether phylogenetic or functional structure or temperature differed significantly across different regions. Similarly, all pair-wise differences were interpreted using Tukey’s HSD post hoc tests. In addition, the correlations were computed using Pearson’s correlation coefficient.

These analyses were performed with R package picante, vegan, phyloch and multcomp in R version 3.2.2.

## Electronic supplementary material

Supplementary information
