# Supplementary material for: Phylogenetic and Functional Structure of Wintering Waterbird Communities Associated with Ecological Differences
Source: Sci Rep. 2018 Jan 19;8:1232. doi: 10.1038/s41598-018-19686-3 (PMC5775246; doi:10.1038/s41598-018-19686-3)
Supplement: Supplementary file 1 — Supplementary information [file 41598_2018_19686_MOESM1_ESM.pdf]

Phylogenetic and Functional Structure of Wintering Waterbird Communities Associated with Ecological Differences

Xianli Che[1, 2, 3], Min Zhang[3], Yanyan Zhao[3], Qiang Zhang[3], Qing Quan[3], Anders Møller[4], Fasheng Zou[3*]

1 South China Botanical Garden, Chinese Academy of Sciences, Guangzhou, China,

2 University of Chinese Academy of Sciences, Beijing, China,

3 Guangdong Key Laboratory of Animal Conservation and Resource Utilization, Guangdong Public Laboratory of Wild Animal Conservation and Utilization, Guangdong Institute of Applied Biological Resources,

4 Ecologie Systématique Evolution, Université Paris-Sud, CNRS, AgroParisTech, Université Paris-Saclay, F-91405 Orsay Cedex, France

*Correspondence to FZ

Email: 610658179@qq.com, chexianli13@mails.ucas.ac.cn

Tel.: +86 020-84182827, +86 18520227531

Fax.: +86 020-84182827

Word count: 5499

Running headline: Che et al.: Phylogenetic and Functional Structure of Waterbird Communities

Supplementary information

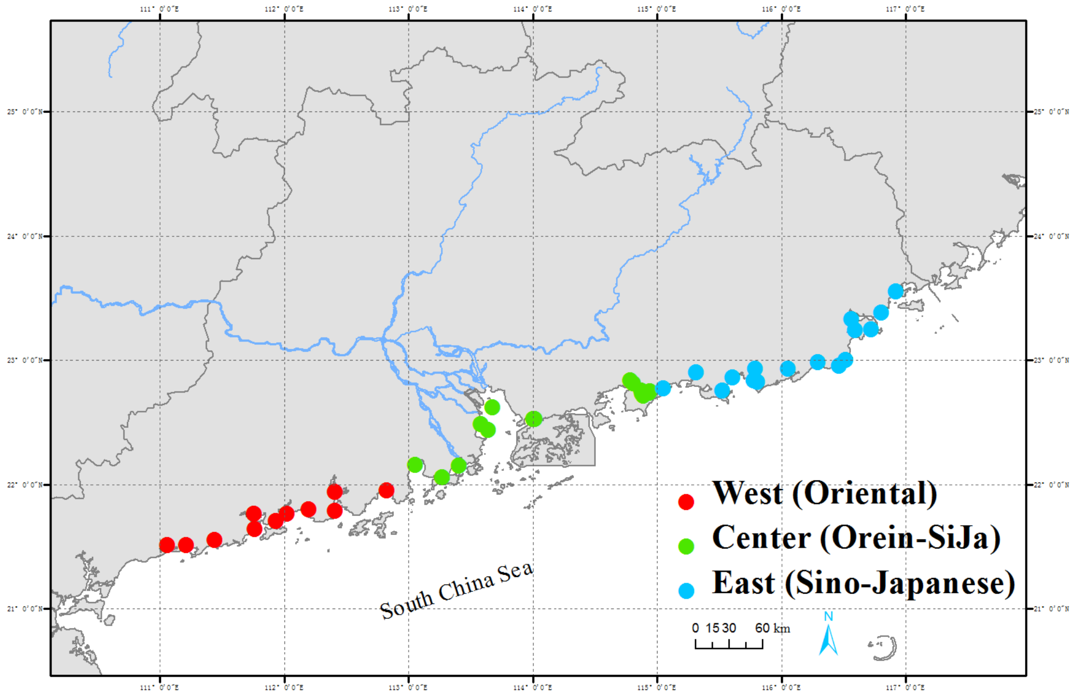

Figure S1 Map of geographical regions in southern China created by ArcMap (V10.0). Geographical regions are labelled by point colures. The red points indicate the study sites of west (Oriental), the green points indicate the sites of center (Orien-SiJa) and the blue points indicate the sites of east (Sino-Japanese）, respectively.

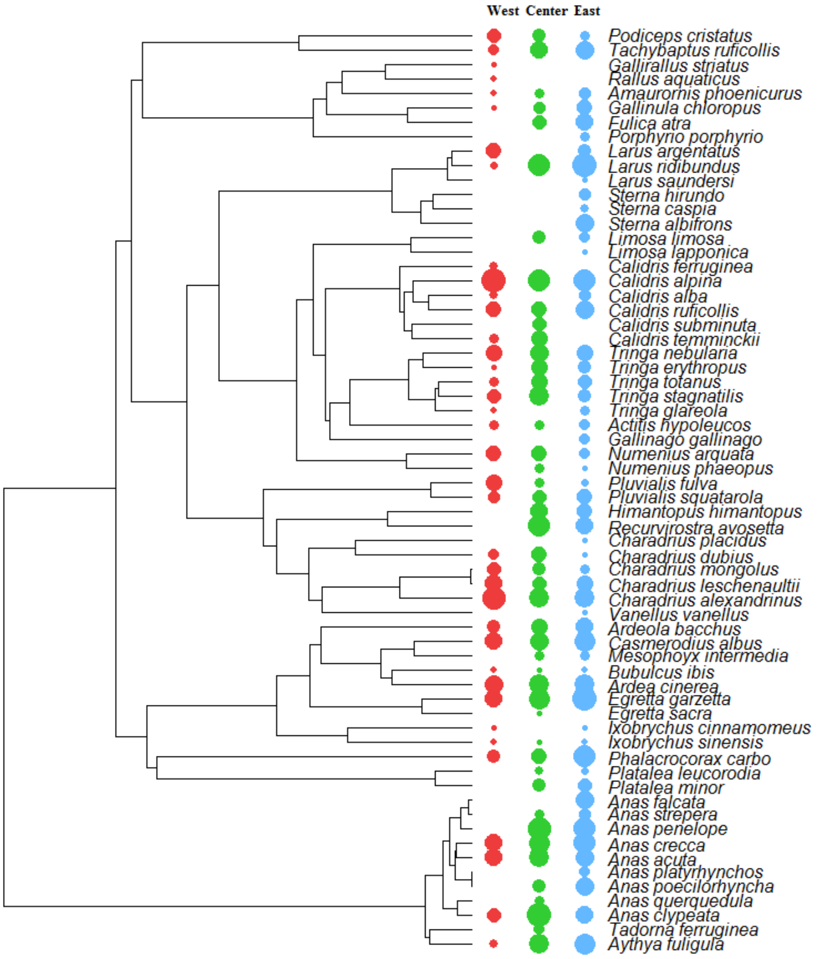

Figure S2 Species composition and their relative abundance across regions. Graphical presentation of the relative abundance of all 64 species across the phylogenetic tree. The red points indicate the abundance in the west, the green points indicate the abundance in the transection zone (center) and the blue points indicate the abundance in the east, respectively.

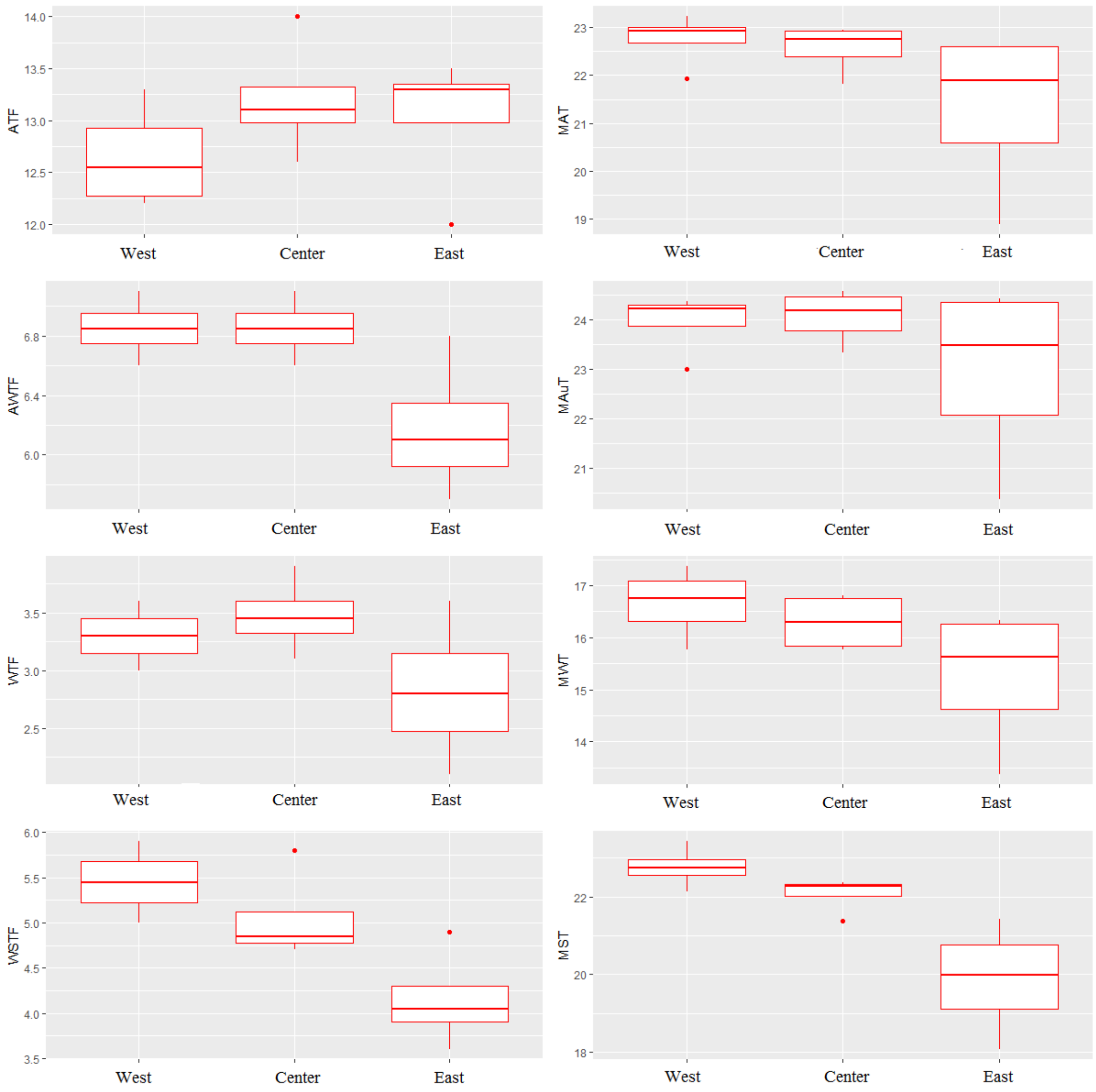

Figure S3 Temperature factors and their difference across regions. Pair-wise differences: for WSTF, $P_{adj/SJ\text{-}Or}<0.01$; for AWTF, $P_{adj/SJ\text{-}OS\ and\ SJ\text{-}Or}<0.05$; for MST, $P_{adj/SJ\text{-}OS}<0.05$, $P_{adj/SJ\text{-}Or}<0.01$.

Table S1 Significance of phylogenetic structure for each measure of community strcture.

	SES (MPD)	SES (MNTD)	D′pw	D′nn
west-center	**0.04**	0.09	**0.001**	**0.02**
east-center	0.60	0.62	**0.001**	0.10
east-west	**0.003**	**0.01**	**0.001**	0.55

Table S2 Strength and significance of phylogenetic signal for community traits.

Traits	Blomberg’s *K*	*P* values
Body mass	0.55	0.001

Beak length	0.68	0.001
Wing length	0.43	0.001
Tarsus length	0.78	0.001

Table S3 Significance of functional structure for each measure.

	Trait SES (MPD)	Trait SES (MNTD)	Trait D′pw	Trait D′nn
west-center	0.98	0.27	**0.003**	0.95
east-center	0.67	0.99	0.13	0.18
east-west	0.59	**0.01**	**0.001**	0.19

Table S4 Significance of Mantel test and partial Mantel test for each measure.

	Mantel test		Partial Mantel test	
	r	*p*	*r*	*p*
TD- SES (MPD)	0.62	0.001		
TD - SES (MNTD)	0.63	0.001		
TD - Trait SES (MPD)	0.40	0.001		
TD - Trait SES (MNTD)	0.59	0.001		
SES (MPD) - Trait SES (MPD)	0.35	0.001	0.14	0.08
SES (MNTD) - Trait SES (MNTD)	0.75	0.001	0.61	0.001
